# Current Approaches in Supersecondary Structures Investigation

**DOI:** 10.3390/ijms222111879

**Published:** 2021-11-02

**Authors:** Vladimir R. Rudnev, Liudmila I. Kulikova, Kirill S. Nikolsky, Kristina A. Malsagova, Arthur T. Kopylov, Anna L. Kaysheva

**Affiliations:** 1 Biobanking Group, Branch of Institute of Biomedical Chemistry “Scientific and Education Center”, 109028 Moscow, Russia; v.r.rudnev@gmail.com (V.R.R.); likulikova@mail.ru (L.I.K.); glucksistemi@gmail.com (K.S.N.); a.t.kopylov@gmail.com (A.T.K.); kaysheva1@gmail.com (A.L.K.); 2 Institute of Theoretical and Experimental Biophysics, Russian Academy of Sciences, 142290 Pushchino, Russia; 3 Institute of Mathematical Problems of Biology RAS—The Branch of Keldysh Institute of Applied Mathematics of Russian Academy of Sciences, 142290 Pushchino, Russia

**Keywords:** structural motifs of proteins, helical pairs, experimental methods, databases

## Abstract

Proteins expressed during the cell cycle determine cell function, topology, and responses to environmental influences. The development and improvement of experimental methods in the field of structural biology provide valuable information about the structure and functions of individual proteins. This work is devoted to the study of supersecondary structures of proteins and determination of their structural motifs, description of experimental methods for their detection, databases, and repositories for storage, as well as methods of molecular dynamics research. The interest in the study of supersecondary structures in proteins is due to their autonomous stability outside the protein globule, which makes it possible to study folding processes, conformational changes in protein isoforms, and aberrant proteins with high productivity.

## 1. Introduction

Simple structural motifs consisting of several elements of secondary structure with unique polypeptide chain folding are objects drawing attention. The interest is raised due to the uniqueness of these structures and their ability to be embryos in protein folding [1]. When modeling a protein structure or predicting its tertiary structure, motifs can be a starting point in searching for possible folds of polypeptide chains, or used as stable structures in protein studies. Efimov et al. presented a classification of structural motifs consisting of α-helices and β-strands having unique folds [1].

The most common structural motifs in homologous and non-homologous proteins are α-α-corner, β-β-corner, α-α- and β-β-hairpins, β-α-β-motif and 3β-corner [2]. The α-α-corner is arranged by two α-helices, which are connected by the polypeptide chain. This is a compact spatial structure with a hydrophobic core and a polar shell. Side chains of residues completely buried in a hydrophobic core are hydrophobic [2]. The β-β-hairpins and β-β-corners can be referred to as β-strands containing supersecondary structures. The β-β-corner can be thought as a long β-β-hairpin folded upright towards itself, so strands rotate to the right around an imaginary axis as they move from one layer to another. The β-β-hairpin, which organizes the helical coil structure or β-β-corner, is right-handed when viewed from the concave side [3]. The β-α-β-motif is a mixed type of SSS (supersecondary structure) [3]. This motif is more complex in terms of structural organization compared to α-α- and β-β-hairpins, and consists of two parallel β-strands connected by an α-helix. Connection between helixes can vary greatly in length and the axis of helix is roughly parallel to the β-strands; thereby all three elements interact to form a hydrophobic core.

Among all known proteins, many small proteins consist of only one or two known structural motifs. This indicates that such structural motifs are autonomously stable [4]. The stability of supersecondary structures was indirectly shown in 1993 by Canadian researchers F. Tsai and J. Sherman (University of British Columbia, Canada) in an experimental study using the circular dichroism method [4]. In this study, the authors showed, using the example of a synthetic horse methemoglobin peptide (residues 80–108) with α-α-corner folding, that the conformation is stable autonomously, outside the protein structure. Thus, in water, the peptide forms a moderately helical shape and acquires a form close to its conformation in the protein in the trifluoroethanol solvent, which mimics the hydrophobic environment of the peptide in the intact protein molecule [4].

In previous studies [5,6], a hypothesis about the autonomous stability of structural motifs in computational molecular dynamics (MD) experiments was proposed and tested. In these works, the trajectories obtained using molecular dynamics were studied in detail from the point of view of the phenomenon of stability, and it was shown that α-α-corners with a short connection are autonomous structures that are stable in an aqueous medium. Similar justifications for the stability of SSS for β-β-hairpins and β-α-β-motifs have also been successfully carried out [7,8].

## 2. Structural Organization of Proteins

Proteins are the most abundant macromolecules in living cells and are found in all cell compartments [9]. The types of proteins are diverse and several thousand in number, each of which is different in size, shape, and biological function [10]. The properties and functionality of a protein depend on its primary structure (sequence of amino acid residues), as well as its spatial organization (tertiary structure, and in some cases, quaternary structure).

### 2.1. Levels of Structural Organization

Proteins usually form compact three-dimensional structures. The structural and functional properties of proteins are determined by the physicochemical properties of the polypeptide chain. The classification of levels of protein structural organization includes primary, secondary, tertiary, and quaternary structures.

The primary structure is determined by a linear sequence of amino acid residues in the polypeptide chain covalently linked to each other by a peptide bond. The lengths of the bonds between the atoms of the peptide group (Cα, C, O, and N), the angles between them, and the differences in the polarity of the atoms of the peptide group reflect the distribution of electron density and possible angles of rotation around atomic bonds, the so-called torsion angles.

Due to the wide range of methods for sequencing the genomes of living systems, the amino acid sequence has been established for most proteins of known organisms. The largest protein knowledge base, including information on protein sequences of known taxonomic groups of organisms, is UniProtKB (http://www.uniprot.org/, accessed on 8 July 2021) [10]. In July 2021, the knowledge base contained 565,254 proteins of various origins (from virus proteins to human proteins).

The polypeptide chain of a globular protein is usually folded into a compact form consisting of fragments with a regular structure. The main regular structures of proteins are α-helices and β-structures. These elements are connected in various combinations by irregular sections of different lengths and conformations, which are also called loops, β-bends, turns, and half-turns.

A generalization of the main geometric parameters of the secondary structure elements is presented in Table 1.

Supersecondary structures (SSS) are a transitional bridge between the secondary and tertiary levels of protein structural organization. Supersecondary structures include elements of the secondary structure, linked by a connection and characterized by a certain spatial geometry [3]. The most common SSS proteins include hairpins formed by α-helix hairpins, β-hairpins, β-α-β, coiled coils, Greek key, α-loop-α, α-turn-α, and Rossmann motifs.

The tertiary and quaternary structures of a protein represent the folding of the elements of the secondary structure of a protein in space. The tertiary structure of a protein is stabilized through chemical interactions as follows:

- Hydrogen bonds between amino acid residues;
- Electrostatic interactions between the side groups of charged amino acids;
- Hydrophobic interactions between side groups of hydrophobic amino acids;
- Disulfide covalent bonds; and
- Interactions with cofactors.

The quaternary structure combines two or more polypeptide chains with a tertiary structure in one protein. Proteins with a complex biological function (e.g., hemoglobin) have a quaternary structure. In some cases, the quaternary structure contains a large number of polypeptide subunits, such as the capsid of the tobacco mosaic virus.

### 2.2. Supersecondary Structure

A spatial structural unit repeated in many proteins or within one polypeptide chain can be considered a structural motif. Structural motifs must be organized by elements of the secondary structure adjacent to the chain. Each type of structural motive is characterized by a certain number and secondary structure elements and a certain mutual arrangement of these elements, both along the chain and in space. Structural motifs of the same type found in different (both homologous and non-homologous) proteins can vary in the length of α-helices, β-regions, and conformations of the irregular connecting regions. However, the general encase of the polypeptide chain in space (that is, the overall stacking of the chain) must remain unchanged.

Globular proteins acquire simple structural motifs, consisting of two sequential regular sections along the chain, and complex motifs, comprising of three or more sections. The most rigorously investigated among the simple ones are α-α- and β-β-corners, α-α- and β-β-hairpins, and L- and V-shaped structures of two α-helices [2,11]. Of the complex supersecondary structures, β-α-β-motifs and 3β-corners are largely scrutinized and have unique spatial folding. The supersecondary structure, also called the structural motif, is a combination of elements of secondary structures. The structural motif reflects the arrangement of atoms in space. 

**Figure 1:**
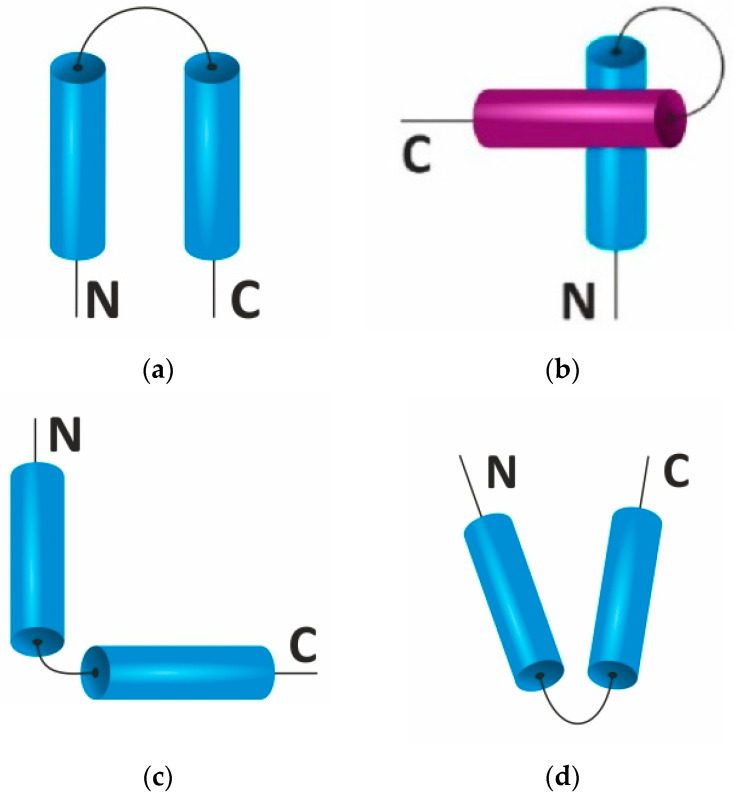
Types of helical pairs containing two α-helices, with a unique folding of the polypeptide chain in space. (**a**) α-helical hairpin, (**b**) α-α-corner, (**c**) L-shaped structure, and (**d**) V-shaped structure. Colors indicate α-helixes lying in different planes (layers).

A sequence motif reflects a specific pattern in a sequence of amino acid residues. Structural motifs containing α-helices are organized by the combination of two or more α-helices and loops or twists connecting them. The difference between “loop” and “turn” is not always obvious, since the length of the loop/turn between α-helices cannot always be used as determining parameter [12]. In this regard, motifs with α-helices are distinguished by the orientation of the helices relative to each other and by their biological functions. For example, helix–loop–helix supersecondary structures are often found in proteins, some of which have important biological functions (Figure 1).

The α-helical hairpin (Figure 1a) consists of two antiparallel α-helices. The loop between the helices in a hairpin can contain two or more amino acid residues. With an increase of the loop length, the allowed number of conformations is increased. When the loop is short, the helices are stabilized relative to each other in space by means of hydrophobic bonds between side chains of amino acid residues. The biological role of the α-helical hairpin is not known, but two α-helical hairpins can arrange a structure of four helices, which, in turn, can organize ligand-binding sites in proteins [13]. Aidan Doherty et al. suggested that one, two, or four copies of the helix–hairpin–helix motif can act as a DNA-binding structure [14]. The authors identified the motif in 14 homologous protein families, including rat polymerase β, endonuclease III, AlkA, and 5’-nuclease domain Taq.pol I. The motifs are structurally similar and, probably, bind DNA nonspecifically through the formation of hydrogen bonds between nitrogen atoms’ protein backbone and phosphate groups of DNA [14].

The α-α-corner (Figure 1b) contains α-helices, which are packed orthogonally (or obliquely relative to each other) and linked by a connection. The corner of the loop is formed by a hydrophobic amino acid residue. The biological role of this type of supersecondary structure is not fully understood [13]; however, the α-α-corner is found in many DNA-binding proteins. Previously, we have noted that post-translational modification of proteins, i.e., phosphorylation of serine and threonine and acetylation of lysine, might be specific to oncological diseases [15,16]. In certain cases, modified amino acid residues are localized in supersecondary structures of the α-α-corner type. We turned special attention to the high stability of α-α-corner motifs, which permits liberation of such motifs from the protein structure for molecular dynamics study. Tsai and Sherman indicate that the α-α-corner motif is found in a large number of proteins and is likely to initiate protein folding. Using the circular dichroism method, high stability and ability to initiate protein folding has been confirmed on the methemoglobin motif of 80–108 residues in a length [4].

Meanwhile, V- and L-shaped structures are often found in ligand-binding proteins, such as calcium-binding proteins (Figure 1c,d). For example, parvalbumin is a muscle protein containing three helix–loop–helix motifs, two of which bind calcium ions. The calcium ion is stabilized by the coordination chemistry between the sidechain carboxylic groups in the loop region of two helices.

α-α-Hairpins are widespread in globular proteins; some proteins mainly consist of such a motif. Hairpins differ in the length of helices, and the between-helices connection. The connection refers to the disordered part of the molecule and, each amino acid within can have a conformation from the allowed areas on the Ramachandran map. The angle Ω between the axis of helices can vary within small limits. α-α-Hairpins can be either right-handed or left-handed, depending on the relative position of the polypeptide chain regions. Globular proteins are tightly packed and have a hydrophobic core surrounded by a polar shell, and α-helical hairpins included in their structure should be formed following these principles. So, one side of the hairpin must be hydrophobic and the other hydrophilic if the hairpin is on the surface of the protein. A hairpin can have both sides hydrophobic if it is completely immersed in the hydrophobic core. To deploy a hydrophobic surface of the α-helix, the hairpin organizes a side-by-side packing according to the “protrusion-into-cavity” principle. In most cases, especially if helices are long enough, the protrusions in the cluster of one helix correspond to the trough of the other, thereby ensuring a dense packing of the structure [17].

**Figure 2:**
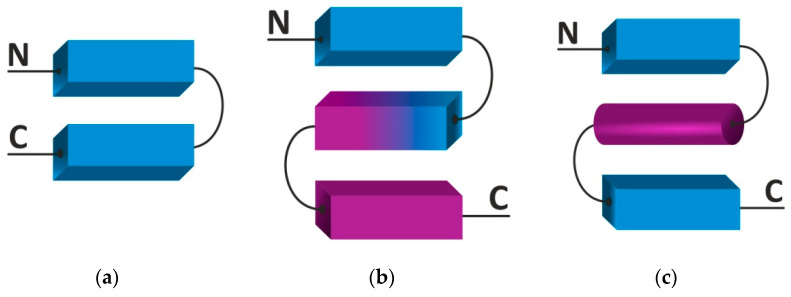
Types of supersecondary structures containing two β-strands, with unique polypeptide chain folding. (**a**) β-hairpin, (**b**) 3β-corner, (**c**) βαβ motif. Colors indicate elements of the secondary strictures lying in different planes (layers).

One of the most widespread in the SSS inflow is the β-hairpin (Figure 2a) [3]. The motif consists of two antiparallel oriented β-sheets linked by a short connection of one to five amino acid residues [18]. The motif is found in the three-dimensional structures of enzymes, carrier proteins, antibodies, and viral envelope proteins [19]. Several scientific groups showed that short peptides can fold into a β-hairpin motif in an aqueous environment [20,21]. Synthetic cyclic β-hairpins are attractive peptidomimetics and affinity reagents with high selectivity to the target phosphorylated peptide in silico [22]. 

The 3ß-corner is a structural motif represented as a triple-stranded ß-sheet folded on to itself so that its two ß-ß-hairpins are packed approximately orthogonally in different layers and the central strand bends by nearly 90° in a right-handed direction when passing from one layer to the other (Figure 2b) [23]. When viewed from their concave surfaces, all 3ß-corners observed can be considered as Z-like β-sheets, i.e., the first and second strands organize a right-turned ß-ß-hairpin and the second and third strands a left-turned ß-ß-hairpin. The 3β-corners are widespread in both homologous and non-homologous proteins and positioned at the edges of domains [24].

The βαβ motif is most often found in the α/β class proteins (Figure 2c) [25]. The motif is formed by two parallel β-sheets linked by an α-helix and stabilized hydrogen bonds, and constitutes functional and active sites (including nucleoside binding (ADP, FAD, NAD) in various proteins [26]. In proteins with dehydrogenase activity, two successive βαβ motifs shape the Rossman fold [27]. In general, it is worth noting that double-stranded supersecondary structures are quite stable and, probably, can be used as a seed in protein folding. 

Typically, proteins containing β-motifs are soluble, whereas β-hairpins, 3β-corners and other SSS containing β-strands are prone to aggregation due to hydrophobic interactions and hydrogen bonding. In this regard, terminal sites of such SSSs are screened by large unstructured loops or charged amino acid residues that provide electrostatic repulsion of hydrophobic β-strands’ nuclei. In addition, the design of β-hairpin forms is a right screw by twisting, which limits possible interactions with adjacent β-strands [28].

### 2.3. Methods for Experimental Analysis of the Secondary Structure of a Protein

Modern analytical approaches enable the experimental detection of secondary structure elements in a protein. The most popular method for studying the three-dimensional structure of a protein molecule is X-ray diffraction (XRD) analysis. The methods still prevails in structural biology and contributes most of the structure entries into the protein databank (PDB) with a wide margin from other experimental methods (cryoEM, NMR) [29]. To date, more than 150,000 spatial protein structures have been identified by X-ray diffraction analysis [30]. XRD provides a resolution of less than one angstrom (1Å) and numerous structural models with a subatomic resolution are now available in the PDB, including rubredoxin (0.68 Å, 2DSX) [31], aldose reductase in complex with NADP+ and the inhibitor IDD594 (0.66 Å, 1US0) [32], high-potential iron-sulfur protein from (0.70 Å, 3A38) [33], small protein crambin (0.48 Å, 3NIR) [34], and triclinic lysozyme (0.65 Å, 2VB1) [35]. The complexity of obtaining a protein crystal is the main limitation of X-ray structural analysis. Furthermore, collecting the X-ray diffraction data and building a three-dimensional structure are painstaking and time-consuming processes.

In the past three decades, methods for solving the spatial structure of proteins using nuclear magnetic resonance (NMR) have been developed. NMR makes it possible to analyze the protein structure in solution under conditions close to native ones in comparison with X-ray structural analysis [36,37]. Moreover, NMR allows the determination of non-rigid regions of a protein as a probabilistic distribution of their coordinates, which is especially important in determining the structures of membrane proteins. Membrane proteins, which account for approximately 30% of the human proteome, assume their native conformation only under the conditions of a lipid bilayer; therefore, obtaining crystals is difficult [38].

NMR also has limitations, and the most critical one is the ability to study the spatial structure of small proteins, i.e., those less than 25 kDa [39]. Moreover, the resolution of NMR analysis is inferior to that of XRD and averages 1.5 Å. The final atomic coordinates of the protein or its secondary elements are also stored in the PDB and account for approximately 10,000 protein structure entries (Table 2) [40].

In addition, there is a set of small proteins annotated in the PDB, which are comprised exclusively of three elements of secondary structures and resemble supersecondary motifs in their three-dimensional structure. This set includes 322 proteins (1a7w, 1b2e, 1dph, 1gjt, 1ij0, etc.) organized by three alpha-helices, 97 proteins (1b13, 1cre, 1fhh, 1io6, 1jbd, 1kbe, 1p9g, 1qh2, 1r0f, etc.) shaped by beta-strands, and 80 proteins of mixed secondary structure elements (1b4o, 1d5q, 1g6x, 1jv9, 1k51, 1nag,1px9, 2ab3, etc.). The existence of such small proteins, structurally close to SSS, is evidence of the autonomous stability of supersecondary structures.

The high-throughput circular dichroism (CD) method plays an important role in determining the secondary structure [41,42]. The CD method aims to identify conformational changes of proteins’ secondary structure, globule stability due to mutations (amino acid substitutions), and protein interactions. One of the advantages of CD is the small amount of protein required for preparation (less than 20 µg per measurement) compared to NMR and XRD, consuming 200 µg or more of protein [13,37]. The CD method is based on the detection of differences in the absorption of right- and left-handed light in helices of different twists. Differences in the absorption of plane-polarized light are translated into elliptically polarized light [40]. However, unlike XRD or NMR, the CD method does not provide information on the coordinates of specific amino acid residues [37].

Cryoelectron microscopy (cryoEM) is another experimental method purposed for the determination of proteins’ 3D structure. In 2017, the Nobel Prize in Chemistry was awarded to Jacques Dubochet, Joachim Frank, and Richard Henderson for their work on cryogenic electron microscopy (cryoEM). The method superseded the protein crystallization procedure, which is especially promising for resolving structures of poorly or non-crystallizable proteins (membrane proteins) [43]. Currently, cryoEM includes single-particle techniques, tomography, two-dimensional crystallography, and electron diffraction by microcrystals (microED) [29]. The first two methods are based on obtaining images of either many identical copies of a molecule (one particle) or one sample at different angles (tomography). The diffraction method traditionally provides the highest resolution of highly ordered single- or multilayer protein assemblies [44]. Based on the diffraction from highly ordered three-dimensional biomolecular assemblies and using approaches borrowed from the crystallography of macromolecules, microED has extended the cryoEM resolution to sub-angstroms (Å).

Recently, there has been a rapid increase in the number of specific structures of macromolecular complexes using cryoEM in the Electron Microscopy Data Bank (EMDB) (https://www.ebi.ac.uk/pdbe/emdb/statistics_main.html; accessed on 27 July 2021). Most of the resolved protein structures have a resolution of 3–4 Å, while some are determined with a resolution of less than 2 Å [45]. Ka Man Yip’s work annotated the structure of apoferritin 3 (EMD-9865, 6Z9F) with a resolution of 1.54 Å, where all atoms, including hydrogen atoms, can be visualized [45]. Other protein models with a resolution of less than 2 Å, determined by cryoEM, were deposited in the PDB with the following access codes: glycolyl-CoA carboxylase with bound CoA (1.96 Å, 6YBQ) [46], glutamate dehydrogenase (1.80 Å, 5K12) [47], beta-galactosidase (1.90 Å, 6CVM) [48], adeno-associated virus (1.56 Å, 7KFR) [49], gamma-aminobutyric acid receptor (1.70 Å, 7A5V) [50], and streptavidin (1.77 Å, 7EFD), etc.

### 2.4. Protein Conformational Stability

Today, the classification includes more than 12 types of protein supersecondary structures [3]. Despite many authors noting the key role of SSS in protein folding (folding of the nucleoli), the issue of SSS stability outside the protein globule has not yet been rigorously scrutinized [15,17,51]. The most common methods for studying of conformational stability are molecular dynamics (MD) and the Ramachandran plot. Using a randomly generated set of SSS (PDB DB) from 163 proteins, Léo Degrève showed that about 30% of the amino acid sequence of globular proteins is involved in the organization of SSS [51]. The structural stability of the selected motifs was further confirmed by the molecular dynamics (MD) approach. In our studies, we have also shown the high stability of SSS for 4 types of helical pairs accessed by the MD [15,16,17]. The β-hairpin motif is also characterized by high autonomous stability, which makes this motif popular for producing artificial protein structures with desired properties (hydrogels, antimicrobial peptides) [52,53,54,55]. 

**Figure 3:**
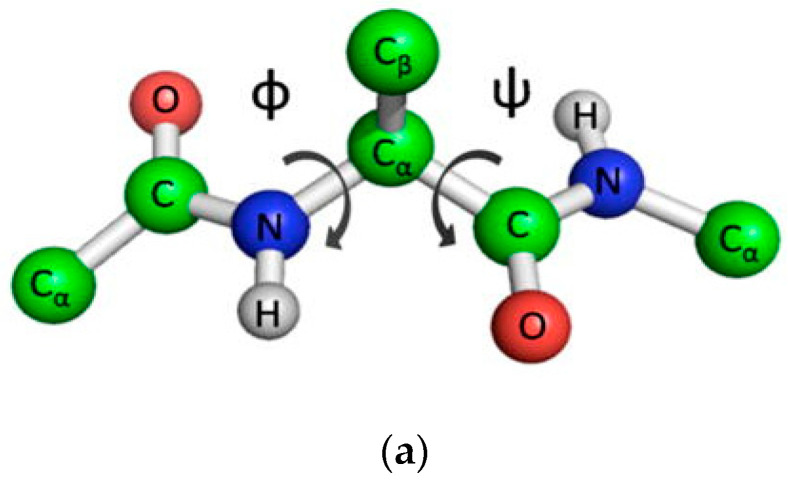

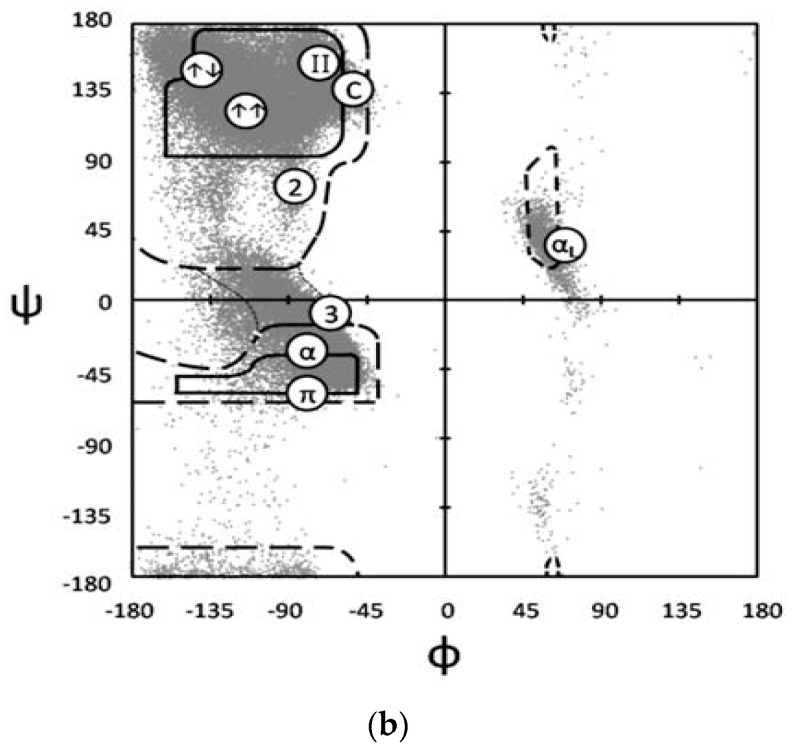
Model of an alanine dipeptide showing the possible rotation planes, which are determined by the torsion angles φ and ψ (**a**). Ramachandran map with highlighted contours of the allowed regions (dashed lines), the core is the most probable conformational states (solid lines), and the extremely admissible limit of conformational states (dotted lines) for the alanine dipeptide (alanine–alanine) (**b**). The gray specks show the 63-149 Ala-like (non-Gly, non-Pro) residues from a diverse set of crystal structures (1.2 A resolution). The zones of conformational states for α-helix (α), 3_10_-helix (3), π-helix (π), left α-helix (α_L_), polyprolium (II), collagen (C), parallel β-sheet (↑ ↑), and anti-parallel β-sheet (↑ ↓). Figure adapted from [57].

Ramachandran’s map has been one of the central concepts in structural biology over the past 60 years [56]. Conformational analysis of proteins using torsion angles remains practically unchanged and is an integral tool in structural biology [57]. One of the basic rules for constructing a map of forbidden and allowed conformations of amino acid residues is to consider atoms as impenetrable spheres [58]. In this version, it becomes possible to determine the conformations of alanine-like amino acid residues (except for glycine and proline), which can occupy one of three “allowed” regions in space (see Figure 3). These include two large regions known as the α- and β-regions for the conformation of α-helices and β-strands, respectively, and a much smaller αL-region, which is a mirror image of the main conformations of the α-region. “Allowed” areas are highlighted in Figure 3 with a dashed line. In these regions, the atoms of the peptide do not experience steric hindrance [59,60].

The construction of maps of forbidden and allowed conformations of more than 150,000 amino acid residues experimentally obtained using X-ray structural analysis of protein structures with a resolution of 1.2 Å and less, confirmed the validity of Ramachandran maps [61,62]. Most of the observations fit into three main groups located in the α-, β-, and αL-regions (Figure 3). To date, Ramachandran maps have been used for stereochemical assessment of the quality of resolved crystal structures in the ProCheck [63] and MOLEMAN2 [64] programs, and in the newer MolProbity [65] program.

## 3. Protein Families

The three-dimensional structure of the protein contains a limited set of folding nuclei, which can be admitted as structural motifs with unique chain folds [1]. Eight types of root structural motifs are currently described: α-α-corner, 3β-corner, s-like β-sheet, z-like β-sheet, 5-segment α/β-motif, 7-segment α/β-motif, abcd-unit, and abCd unit [66]. SSS are universal for various proteins, regardless of their origin and homology [1]. They can also be used as initial structures in protein modeling [67,68]. 

The availability of structural information about proteins supported the development of several structural classifications:

- SCOP (structural classification of proteins) [69],
- PCBOST (protein classification based on structural trees) [70,71],
- PROSITE (database of protein families and domains) [72], and
- CAZy (carbohydrate-active enzymes) [73];
- CATH (classification of protein structures) [74].

Early work on protein structures’ classification revealed regularities between the content of secondary structure elements in proteins [75] and protein topology [76,77,78]. Such patterns became apparent when scientists Ptitsyn O.B. and Finkelstein A.V. created a new direction in structural biology—the physics of proteins [79]. The most extensive bibliography of studies on the structural classification of proteins and the structural determinants of proteins is presented in the SCOP database (http://scop.berkeley.edu; accessed on 4 August 2021) [80,81].

The SCOP database contains the following protein classes and their composition (including SSS):

- α proteins (46,456 protein structures and 289 folds);
- β proteins (48,724 protein structures and 178 folds);
- α/β proteins (51,349 protein structures and 148 folds);
- α + β proteins (53,931 protein structures and 388 folds);
- multidomain α and β proteins (56,572 protein structures and 71 folds); and
- membrane proteins, surface proteins, and peptides (56,835 protein structures and 60 folds).

The structural classification of proteins presented in the SCOP reflects the hierarchy of protein structures through the analysis of evolutionary and structural similarities. 

Another method for the classification of proteins was recently developed by the group of A.V. Efimov (Institute of Protein Research of the Russian Academy of Sciences). Taking into account supersecondary structures as folding nuclei, the method is based on the spatial similarity and the generality of protein folding pathways [2]. This classification, namely PCBOST, is based on the structural trees of proteins, and not on the similarity of their evolution and biological function. The structural tree of a protein is a set of allowed intermediate and final spatial structures that can be obtained from the starting structure by the sequential adding (or extension) of other elements of the secondary structure. Currently, the PCBOST classification has been developed for 18 structural protein trees (according to the PCBOST web service, http://strees.protres.ru/help.htm; accessed on 9 August 2021).

Supersecondary structures have a unique spatial folding of polypeptide chains. As a rule, α-helices and/or β-strands in a supersecondary structure have the same location wherever these structures are found, regardless of whether proteins are homologous or not. It has been shown that the α-α-corner structure is found in proteins more frequently. The first and second helices in the α-α-corner are usually referred to as the A and B helices, respectively. A short bridge between the helices allows their arrangement in orthogonal orientation. However, regardless of the length and conformation of the connection, α-α-corners almost always organize as a left superhelix.

The amino acid sequence corresponding to the α-α-corner with a short connection can be characterized by the following features:

1. Hydrophobic amino acid residues should be at positions essential for the organization of hydrophobic clusters of A- and B-helices.
2. There should be a 1-3-8 gap between clusters.
3. The last position of the A-helix should be occupied by small or flexible residues, such as glycine, arginine, and lysine.
4. The first position of the B-helix should be occupied by a polar uncharged amino acid residue (according to the Kyte–Doolittle scale [82]) or residue with small side group (glycine, alanine, and proline).

Almost all positions necessary for clusters in the A and B helices are hydrophobic and conserved. Polar uncharged serine and threonine are found in invariant clusters because their side groups form hydrogen bonds with the main chain of the polypeptide. Amino acid residues lysine, arginine, glutamine, and glutamate occupy border positions in hydrophobic clusters since their polar NH2-and COOH-groups are remote from the main chain of the polypeptide.

As noted above, α-α-corners are widespread in protein globules [83]. Furthermore, α-α-corners are found in various proteins of almost all known living organisms, for example:

- In cytochrome C, a protein of the respiratory electron transport chain involved in electron transfer;
- In papain, an enzyme of unripe papaya fruit that is used for the development of enzyme-linked immunosorbent assays;
- In hexokinase, which catalyzes the transfer of a phosphoryl group from ATP to glucose;
- In the lambda repressor, a protein that affects transcription from the RM and R promoters.

In summary, the α-α-corner is a structure depending on the rest of the protein molecule and can act as a folding nucleus in the process of folding a domain or a whole protein [84].

## 4. Methods for Bioinformatical Analysis of Protein Structure

**Figure 4:**
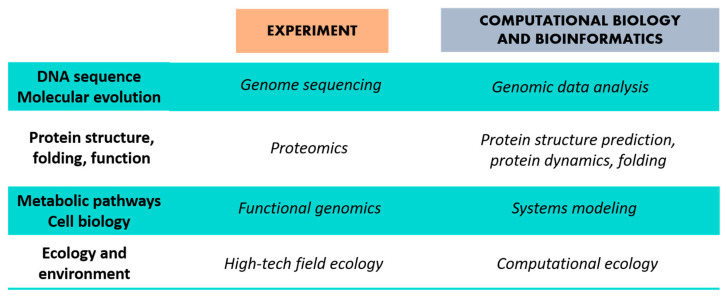
Possibilities of information methods in biology. Figure is adapted from Gibas and Jambeck [85].

Methods of bioinformatic analysis of protein structure are quite extensive due to the availability of experimental data on proteins’ structure (Figure 4). Bioinformatic analysis works under a wide range of scientific problems, which are attained by two main directions:

- Functional tasks → presentation, storage, and dissemination of experimental data.
- Analytical tasks → development of data analysis tools to generate new knowledge.

Functional tasks are associated with the exchange of available knowledge and experimental biological data. The information accumulated to date about proteins is summarized and arranged in public databases and repositories. The adaptation of standard computer science methods for storing and processing of user requests is not a trivial task but involves processing of large amounts of data and operating with rank descriptors. It is necessary if handled data are obtained by multiple research groups, in several technical repeats, using different analytical equipment with different resolutions, and the final results can vary. 

### 4.1. Protein Amino Acid Sequence Databases

Today, researchers have access to various databases of protein amino acid sequences. The variety of databases encompasses repositories of amino acid sequences with rigorously annotated records (UniProtKB, NCBI), including a description of structural elements, post-translational modifications (PTMs), and the biological function of the protein.

The knowledge base UniprotKB (http://www.uniprot.org/; accessed on 10 August 2021) [86] consists of two sections:

- Swiss-Prot—protein database containing manually curated and verified records [87].
- TrEMBL—protein database of automatically annotated entries.

The UniProtKB knowledge base is by far the largest and arranges almost all available information about proteins. This knowledge base realizes cross-references to a numerous tools aimed at processing with amino acid sequences (for example, search for homologs and amino acid sequence alignment, or BLAST) [88].

The NCBI database is popular in biomedical research (National Center for Biotechnology Information, http://www.ncbi.nlm.nih.gov/; accessed on 8 July 2021) [89]. The database is accessible via the Entrez search engine. The NCBI database provides information on protein domain databases, DNA (GenBank) [90], RNA, databases of scientific literature (PubMed) [91], and taxonomic information (TaxBrowser), providing a search for data on a specific biological species (taxonomy). It also contains various standard bioinformatics programs (BLAST). 

The NCBI database (http://www.ncbi.nlm.nih.gov/; accessed on 8 July 2021) was created to solve the following tasks:

- Design of automated systems for data storing and analysis for molecular biology, biomedicine, and genetics tasks;
- Computer processing of biological data; and
- Popularization of databases and software for researchers.

### 4.2. Databases on Protein Structure

Protein DataBank (PDB) is the most important public and accessible database of protein structures. Data on the protein/peptide structures are stored in the text format “* .PDB” and available for use. The “* .PDB” format is comprised of mandatory and optional records, in which calculated coordinates of each atom are presented along with the experimental details regarding the target protein/peptide. To handle PDB files’ visualization and analysis, RasMol [92,93], PyMOL [94] and several other programs have been developed. 

#### Alignment of Amino Acid Sequences

One way to describe the evolution of a protein sequence is to compare sequences of homologous proteins, where homology depends on the sequence of a common ancestor. Using comparative analysis, models of protein evolution were elucidated using the frequency of occurrence of various amino acid residues at specific positions among homolog sequences. Such models enable the detection of point mutations that are fixed in the evolutionary process since they have a neutral meaning or a positive meaning for the biological function of a protein (rare events). Evolutionary models are typically used to identify or align homologous proteins and act as a source of conformation about the protein evolution. The alignment of evolutionarily related amino acid sequences is the most crucial bioinformatic task since it enables the understanding of evolutionary events and pathways. In addition, sequence alignment reveals common features of proteins’ structure or function.

Modern bioinformatics is a powerful tool for amino acid sequence alignment. All alignment methods can be divided into two groups.

- Sequential pairwise alignment of sequences; and
- Multiple alignments.

Pairwise and multiple alignments can be global (for an entire amino acid sequence) or local (for specific regions of the amino acid sequence). Global alignment is indispensable for determining the relationship between proteins, and local alignment is indispensable for identifying conserved regions in the polypeptide chain. The alignment algorithm is defined by two mechanisms:

- A ranking function to assess the effectiveness of the alignment; and
- An alignment strategy with the ability to identify amino acid substitutions, insertions, and deletions.

Many factors are involved in the evolutionary selection and fixation of amino acid mutations. Mutations can be caused by internal factors, such as errors in reading the genetic code at the level of transcription, errors in biosynthesis, and other external environmental factors. To date, the problem of an adequate scheme of searching for mutations within aligned sequences, including of unrelated proteins, remains. The most commonly used alignment involves permutation matrices and is based on statistical observation of amino acid substitutions in homologous proteins.

The use of sequential pairwise alignment methods is limited by the prerequisite length identity of the analyzed sequences. If one of the analyzed sequences is significantly shorter, then the smaller sequence will be supplemented with gaps to equalize their total length. In this case, the alignment results are significantly distorted.

## 5. Methods for Predicting Protein Structure

As has been touched on before, the supersecondary structure is a motif of special geometry, consisting of several elements of the secondary structure. Supersecondary structures are the bridge between the secondary structure and the tertiary structure [3]. Several efficient computational prediction methods for SSS have been recently announced.

Prediction of the protein spatial folding from its amino acid sequence is challenging. There is also a counterpart issue when the prediction of an amino acid sequence with a given three-dimensional structure is of special interest in biotechnology [95]. However, methods for protein structure prediction and design have advanced significantly over the past decade. New algorithms for constructing protein spatial structures are employed to design fluorescently labeled proteins with new or improved properties and to construct signaling proteins with therapeutic potential [95,96].

Currently, two approaches are used to predict the structure: template-based modeling (TBM), in which the known structure of homologous protein is used as a template for the unresolved protein structure; and modeling without a template, which uses energy functions to characterize the most advantageous conformations. These two approaches are not self-excluding and can be combined: for example, prediction of protein structure from a template and subsequent refinement of the conformation using energy functions. Machine learning methods and high performance of modern computing resources encourage the successfully combination of these methods [97]. Both approaches can be used to predict the SSS.

### 5.1. Template-Based Modeling

Template-based modeling (TBM) is based on the observed similarity of the modeled sequence with the empirically characterized (NMR, cryoEM, or X-ray structural analysis) protein structure [98,99]. In other words, if the structure of one protein within a proteins family has been determined empirically, other family members can be modeled based on comparison with the known structure. The PDB database remains a reliable source of templates for predicting protein structure [100]. TBM is based on the fact that a small variation in the amino acid sequence of a protein usually leads to an insignificant change in its three-dimensional structure [101]. The success of TBM is limited to the selection of a homologous template in the PDB. If the evolutionary relationship between the query and the template is distant (the so-called “twilight zone” with homology below 30% between the compared sequences), the prediction accuracy is sharply reduced [100,102]. However, the three-dimensional structure of proteins within one family is rather conservative [103].

The discrepancy between the number of protein sequences (Uniprot/ TrEMBL, more than 55,000,000 records) obtained by virtual translation from annotated genes annotated and the number of structures stored in the PDB database (more than 150,000) is obvious. However, any known amino acid sequence contains at least one domain that can be matched with a template [104]. Thus, exact matching of a template with a request and selection of a template is a difficult task, especially for proteins, where only distant homologs are available [99]. Thus TBM was designed to bridge the gap between the number of amino acid sequences and resolved protein structures [99,105,106].

**Figure 5:**
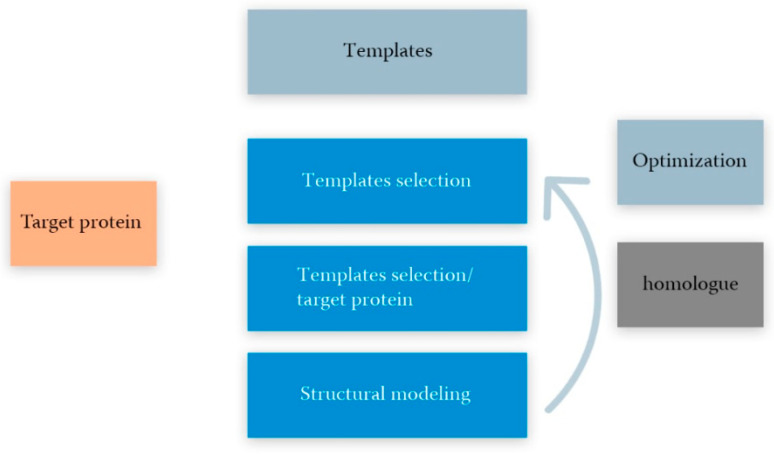
Main stages of comparative modeling of protein structure, including template selection, alignment of template-target protein sequences, modeling of the target protein structure, and quality assessment (optimization).

Comparative modeling usually relies on the knowledge of structure of a homologous protein, which is considered as a template for constructing an unknown target protein with acknowledged amino acid sequence. This process can be divided into several stages (Figure 5) [107]:

- The choice of a template(s) for the sequence of the modeled protein as a query and PDB as a database using a basic local alignment (BLAST, blast.ncbi.nlm.nih.gov, accessed on 20 September 2021);
- Initial alignment and correction of amino acid sequences of the modeling structure and the template(s). Usually performed using the blocks substitution matrix (e.g., BLOSUM80, BLOSUM62 and BLOSUM45) [99];
- Backbone generation, or determination of the structure of conservative areas and structurally variable areas. The stage ends with the construction of a three-dimensional reference structure using a position-specific scoring matrix (PSSM) [108] or hidden Markov model (HMM) [108];
- Copying structurally variable regions of the template(s);
- Construction of structurally variable regions; for example, using CODA runs two programs for the prediction of the structurally variable regions of protein structures: FREAD, a knowledge-based method using a database of fragments taken from the PDB and PETRA, an ab initio method using a database of computer-generated conformers [109];
- Side-chain modeling; for example, using the SCWRL software designed specifically for predicting side-chain conformations taking into account a fixed skeleton derived from the experimental structure of the PDB [110,111]; and
- Model optimization, including optimization of stereochemistry energy minimization, molecular dynamics, and estimation of prediction errors for homologous proteins using the support vector machine (SVM) regression method [112];
- Validation (experiment) is the final step of the theoretical model. Experimental data ranging from ligand binding to spectroscopy or X-ray crystallography can be used for the evaluation. The method for validating a three-dimensional structure of homology according to its experimental analog is the root-mean-square deviation (RMSD), which gives the average value of the distances between all atoms for two three-dimensional structures [107].

As has been mentioned above, the basis of all algorithms for comparative modeling is a successful choice of the most evolutionarily close template sequence. The selection of template sequences is generally carried out automatically by the SWISS-MODEL program in accordance with the following criteria:

- Level of similarity between the target sequence and the template,
- The presence of an experimentally solved structure with high resolution, and
- The presence of ligands or cofactors.

Ideally, the target sequence should be provided with one high-quality template; however, in practice, the template cannot be found for the entire target sequence, but only for a separate structural domain (see Figure 5) [107].

Simulations, like all other stages, are carried out automatically. If the alignment between the target protein and the template shows high identity, a fully automated structural modeling approach could be applied, then the user is required to enter only the amino acid sequence itself or the UniProtKB identifier of the target protein. Typically, this method works for more than 50% of identical sequences. If several available templates are found for the target protein, then the program will select the template with the highest quality score in the “default” mode. If desired, the user can select any other template from the proposed list (semi-automatic mode).

The sequence alignment between the target protein and template can be performed in a semi-automatic mode using several tools: BLAST, PSI-BLAST, and HMM-HMM.

In turn, PSI-BLAST offers templates with less sequence identity to the target protein. The selectivity and sensitivity of the search can also be adjusted by changing the e-value threshold. This method increases the chance of proper pattern detection, but the proportion of false-positive patterns would also increase [113].

Validation of the obtained structural model is one of the most important steps in this algorithm, as the quality of the model determines the biological capabilities of the protein and depends on the evolutionary distance between the target protein and the template. In addition, TMB programs generate a large number of three-dimensional protein models and rank them according to different assessment methods. A more reliable result can be provided by combination of several assessment methods, divided into several groups.

First, methods based on the calculation of force field parameters, or a set of standard parameters and equations that describe bond lengths, bond angles, dihedral angles, improper planes, electrostatic and van der Waals forces, and optimal stereochemistry. 

For this purpose, researchers use the MolProbity web service, as a model checking system for protein and nucleic acid structures (http://molprobity.biochem.duke.edu; accessed on 2 July 2021) [114]. The service is based on previously developed systems such as the PROCHECK [63] and WHATCHECK [115], which calculate the conformations of amino acid residues and account for structural functions (bond lengths and torsion angles). The MolProbity service allows for contact analysis of all atoms, including hydrogen atoms (atomic conflicts), analysis of allowed conformational states of amino acid residues using Ramachandran maps, and Cβ-rejection criteria (backbone emissions) [114].

Second are methods for evaluating the interaction energy. For example, the QMEAN algorithm is a composite estimate using the statistical potentials of the Cβ interaction, the pair energy of all atoms, the torsion angle energy, and the solvation energy [116]. 

Further, a machine learning-based approach is essential for the predicting of errors in homologous models and employs a support vector machine (SVM) regression method [112]. The deep residual neural network ThreaderAI is also widely utilized for model improvement [99]. The model uses deep learning to predict the residual-residual alignment probability matrix by integrating the sequence profile, predicted sequential structural features, and predicted residual–residual contacts for the subsequent pattern-simulated structure matching by applying a dynamic programming algorithm to the probability matrix [99]. The NDThreader (new deep-learning threader) method is also used to solve TBM problems [99] and uses DRNFs (deep convolutional residual neural fields) to match the template/modeled protein request, and ADMM (variable direction multiplier method) and DRNF to improve template/modeled protein alignment by exploiting predicted distance potential. 

The final stage of TBM is experimental validation of the theoretical model. Experimental data from a variety of analytical measurements from ligand binding detection to spectroscopy or X-ray crystallography can be used. The comparative analysis of similarity between the empirical and simulated protein structure can be performed by estimating the root mean square deviation (RMSD) of the distances between all atoms, the mean distance between the Cα atoms, scaled by the template modeling distance parameter [117], the similarity of interatomic contact areas’ (all atoms or their subsets) contact area difference score (CAD-score) [118], and other points of estimate.

### 5.2. Template-Free Modeling

Protein structure modeling without the use of templates can be applied to proteins without analyzing the global structural similarity to proteins in the PDB database. In the absence of a structural template, this approach requires a strategy for the selection of conformational samples to create probable models and ranking criteria [95]. The patternless structure prediction process can be described in four steps. In the first stage, multiple alignments of the sequences of the simulated protein and target sequences are constructed. Further, target sequences are used to predict local structural features, such as secondary structure and twisting angles of the main chain, possible interactions of amino acid residues, etc. For example, PSIPRED Protein Analysis Workbench is a world-renowned web service providing a diverse toolset for the prediction and annotation of proteins, including predicting the secondary structure of a protein based on position-dependent scoring matrices (PSIPRED 4.0), predicting disordered regions with annotated protein binding activity (DISOPRED3), prediction of helix transmembrane packing location using residual contacts and directional action algorithm (MEMSAT-SVM), combination of coevolution methods for accurate prediction of contacts and long-range hydrogen bonds in proteins (MetaPSICOV 2.0), prediction of hot spot remnants at protein–protein interfaces using support vector machines (HSPred), and other services [119].

Libraries of protein backbone fragments can also be integrated into the model building. Prediction of the local structure and contacts aids in the construction of a 3D model using gradient-based optimization, distance geometry, or fragment reassembly methods [95]. The 3D model is usually constructed with inaccurate detail and requires subsequent refinement using the energy function of all atoms, identifying clusters of similar low-energy conformations, from which the representative model is selected as the final prediction.

Template-free modeling reconstructs the protein structure from three to nine fragments extracted from proteins annotated in the PDB database [120]. Such fragments are selected with the similarity of local sequences and the similarity between the known and predicted secondary structure, and then assembled using the search strategy with simulated Monte Carlo annealing [120]. Another method for fragments assembling is C-QUARK, which integrates multiple deep-learning and coevolution-based contact maps to guide the replica-exchange Monte Carlo fragment assembly simulations [121].

The non-template approach has more scope for modeling the target of a new style that is not typical for templates. However, proteins with a length of more than 150 amino acid residues still present a major challenge for non-template modeling methods due to the exorbitant computational requirements and low accuracy of the force field. The prediction of the contact map based on the coevolution approach has recently demonstrated the promise of overcoming this limitation of the folding length of structures ab initio [121].

The first computational method, AlphaFold, is becoming a revolutionary approach and is designed to predict the protein structure with atomic precision, even if a pattern cannot be detected [122]. In most cases (95%), this computational approach predicts protein structures with an accuracy close to the experimental one. The AlphaFold neural network is included in the CASP14 assessment (critical assessment of methods of protein structure prediction, May–July 2020; AlphaFold2) [123]. 

The high accuracy of AlphaFold prediction is due to the inclusion of new neural network architectures and learning approaches based on the evolutionary, physical, and geometric constraints of protein structures. To obtain an accurate and precise domain structure (average backbone accuracy of 0.96 Å), AlphaFold can create highly accurate sidechains when the backbone is highly precise and significantly improved over the template-based method, even when reliable templates are available. The full atomic accuracy of AlphaFold can achieve 1.5 Å with a standard deviation (CI 95%) within a 1.2–1.6 Å range versus accuracy of 3.5 Å with a standard deviation (CI 95%) of 3.1–4.2 Å among other alternative methods. Finally, the model is capable of providing accurate estimates of its reliability from residuals, which should ensure that these predictions are used with confidence [122].

### 5.3. Algorithms for Predicting Some Supersecondary Structures

In the prediction of supersecondary structures, some TBM methods, including MODELLER [124], ModBase [125], I-TASSER [125], Rosetta [126], and QUARK [127], can be utilized. Homology modeling is a reliable method for predicting the structure of a protein molecule from an amino acid sequence. The disadvantage of this method is the need for an experimentally established tertiary structure of the protein that is closest to the amino acid composition.

The researcher has access to specialized services designed for specific types of supersecondary structures of proteins; for example, the predictors the SpiriCoil, LogiCoil, and MultiCoil2 predictors predict only coiled coils [128]. The support vector machine algorithm can be used to identify the β-hairpin in enzymes, where it participates in the formation of ligand binding sites [129]. The chemical shift function and quadratic discriminant analysis of experimental NMR data are robust algorithms for predicting the beta hairpin [130]. The HTHquery web service (http://www.ebi.ac.uk/thornton-srv/databases/HTHquery; accessed on 21 July 2021) can be used to predict helical pairs. This TBM takes into account the availability of a putative structural motif and the positive electrostatic potential of the immediate environment of the SSS. The score set is calculated based on each template using a linear predictor [131].

The prediction of the βαβ-motif, a structure that is more complex than those described above, can be carried out using the support vector machine algorithm [26]. The algorithm takes into account the amino acid composition and the position of amino acids in the motif, information on the secondary structure of amino acid residues. StackSSSPred (from the English “stack supersecondary structure prediction”) is a specialized tool for predicting supersecondary structures from a sequence, based on machine learning [3]. The creation of specialized methods for predicting individual types of SSS seems to be a promising direction in the field of protein engineering. Small and simple protein structures with desired properties can be obtained by de novo protein design [128].

## 6. Study of the Geometric Parameters of Supersecondary Structures in Proteins

Molecular dynamics modeling (MD) has been used by researchers to study the folding dynamics of peptides and small proteins, their stability, and their biomolecular aggregation [132].

In calculating the MD force fields, special attention is paid to the consistent and correct parameterization of atomistic interactions. Research continues to improve the accuracy of the force fields AMBER [133], CHARMM [134], GROMOS96 [135], and OPLS [136] by refining the parameters of the torsion angles of the protein backbone and achieving conformational equilibrium between extended and helical structures [132]. In the study by Manuel Rueda, a comparative analysis of the force fields AMBER, CHARMM, GROMOS96, and OPLS [137] was carried out, and similar results were obtained for 30 protein structures under conditions close to the native ones.

### 6.1. Revelation of Supersecondary Structures

The secondary structure is a key element in the architectural organization of proteins. Accurate determination of secondary structure elements is a crucial step in the analysis and modeling of the protein structure. Because supersecondary motifs are a collection of secondary structural elements, mathematical algorithms are used to identify such elements, among which the most popular are DSSP [138], DSSPcont [139], STRIDE [140], P-SEA [141], and KAKSI. The defined secondary structure of proteins (DSSP) algorithm determines eight states of the secondary structure based on the analysis of hydrogen bonds with energies below −0.5 kcal/mol, stabilizing the structure. In turn, DSSPcont is a modification of the DSSP for the analysis of probable structural changes by considering the thermal motion of the molecule.

The STRIDE algorithm uses the calculated hydrogen bond energies and rotation angles φ/ψ to determine the secondary structure. The torsion angles were determined according to the area of the Ramachandran map. P-SEA defines the secondary structure based on the coordinates of the Cα atoms. The predictive assignment of linear secondary structure elements (PALSSE) describes three states of the secondary structure in a vector form based on the coordinates of the Cα atoms. STICK finds a set of line segments independent of the definition of the outer secondary structure, which allows the segments to be used as a new base for defining the secondary structure. This is accomplished by determining the average increment along each axis to characterize the segment. In this case, elements of the secondary structure are described by a continuous value and, therefore, are not limited by the usual classes of structures. It allows encoding of structures among the “classical” secondary structures as line segments that can be used in structure comparison algorithms. Finally, the KAKSI algorithm determines the secondary structure based on measurements of the distance Cα of atoms and angles φ/ψ. The algorithm detects bends in spirals.

### 6.2. Analysis of the Geometries of SSS and Tertiary Protein Structures

The main measure for representing the set of φ/ψ angles is the Ramachandran map. The map shows the relationship between angles and the conformation of a protein molecule, allowing the correlation of the amino acid residues to the secondary structure, to track allowed and forbidden conformations. Molecular dynamics is the main method used to model and analyze conformations of protein molecules. Many programs are available today, but AMBER, GROMACS, NAMD, TINKER, OpenMM, CHARMM, and DESMOND are the most applicable for biomolecular modeling. Each program includes the functionality of calculating MD, analyzing modeling data with built-in utilities: torsion angles, hydrogen bonds, conformations, physical and physicochemical characteristics, etc. In addition to built-in utilities, specialized programs have been developed, mainly in Python.

MDTraj is a modern, lightweight, and fast software package for MD simulation analysis. MDTraj reads and writes track data in a wide variety of formats. It provides a wide range of trajectory analysis capabilities, including calculating the minimum standard deviation, assigning a secondary structure, and extracting general order parameters. The package focuses on interacting with the broader scientific ecosystem in the Python programming language, bridging the gap between MD simulation data and the rapidly growing set of standard statistical analysis and visualization tools in Python.

Most of the codes generate output trajectories in their own formats, so the development of new trajectory analysis algorithms is limited to specific user communities, and widespread adoption and further development are delayed. MD analysis solves this problem by abstracting access to the raw MD modeling data and presenting the user with a single object-oriented Python interface. Thus, it allows users to write codes that are portable and immediately usable in virtually all biomolecular modeling communities. The UI and modular design work equally well in complex scripted workflows, as the basis for other packages, and interactive and fast prototyping work in IPython/Jupyter notebooks, especially when combined with molecular imaging provided by nglview and timing analysis of the rows using Pandas. MDAnalysis is written in Python and Cython and uses NumPy arrays for easy interoperability with the wider scientific Python ecosystem.

MDPlot is an R library that can handle the output of various programs and provides plotting functions to automate the visualization of molecular dynamics simulation results. This is especially useful in cases where graph generation is quite tedious because of complex file formats or when many graphs are generated. Supported plots range from standard plots such as RMSD/RMSF (root mean square deviation and root mean square fluctuation, respectively) to less standard plots, such as thermodynamic integration analysis and monitoring of hydrogen bonds over time. Thus, working with data and integrating additional file formats is straightforward. The download functions currently support the GROMOS, GROMACS, and AMBER file formats.

## 7. Conclusions

This paper shows the possibility of analyzing the autonomous stability of structural motifs. The supersecondary structures are compactly organized and stable elements of proteins that can be studied as independent motifs. Dense spatial packing of α-α-corners and high stability are provided by a multitude of hydrogen bonds and van der Waals interactions of polar and charged side radicals with each other and within the immediate environment. The MD study of the supersecondary motif, which is small in terms of the number of atoms, in comparison with the whole protein molecule, is distinguished by high performance, lower requirements for computing resources, and low cost of the experiment.

Isolated supersecondary structures are of interest for the study of structural changes caused by amino acid substitutions and by PTMs. The study of diseases associated with the appearance of modified aberrant proteins (proteinopathies) is the most accelerating direction of structural biology [142]. The most significant breakthroughs are expected in diagnostics of aberrant protein forms of amyloid-beta isomers in Alzheimer’s disease [143], osteopontin b and c splice isoforms in prostate cancer [144], amino acid substitution in protein C7 in type-2 diabetes [145], and amino acid substitution of the Ras protein in pancreatic cancer and colorectal cancer [146]. Post-translational modifications specific to serological proteins in patients with colorectal cancer are localized in helical pairs, including α-α-corners. It is likely that the identified PTMs modulate the biological function of the protein, and that the proteins themselves are associated with oncogenesis [15].

## Figures and Tables

**Table 1:** Geometric parameters of the elements of the secondary structures of proteins.

Structure	Hydrogen Bond	Residue/Coil	Displacement/Residue (Å)	φ	ψ
Right α-helix	CO_o_–HN_+4_	+3.6	1.5	−60°	−45°
Antiparallel β-sheet	between strands	−2.3	3.4	−135°	+150°
Parallel β-sheet	between strands	−2.3	3.2	−120°	+135°

**Table 2:** Composition of annotated protein structures in the Protein Data Bank database (https://www.rcsb.org/stats/growth/growth-xray accessed on 5 October 2021).

Research Method	Total Number of Entries Available	Number of Protein Structures	The Number of Structures of Complexes of Proteins and Nucleic Acids
X-ray	160,277	159,817	9715
NMR	13,500		
EM	8870		
Mixed	193		

## Data Availability

This is a review paper that collected from public data listed in the Reference section.
